# The Influence of Obesity on Puberty and Insulin Resistance in Mexican Children

**DOI:** 10.1155/2018/7067292

**Published:** 2018-09-03

**Authors:** Edith Cardenas-Vargas, Jairo A. Nava, Idalia Garza-Veloz, Mayra C. Torres-Castañeda, Carlos E. Galván-Tejada, Miguel A. Cid-Baez, Rosa E. Castañeda-Arteaga, Yolanda Ortiz-Castro, Perla M. Trejo-Ortiz, Roxana Araujo-Espino, Fabiana E. Mollinedo-Montaño, Jose R. Muñoz-Torres, Margarita L. Martinez-Fierro

**Affiliations:** ^1^Hospital General Zacatecas “Luz González Cosío”, Servicios de Salud de Zacatecas, Zacatecas, 98160 ZAC, Mexico; ^2^Molecular Medicine Laboratory, Unidad Academica de Medicina Humana y C.S., Universidad Autonoma de Zacatecas, Zacatecas, 98160 ZAC, Mexico; ^3^Unidad Academica de Ingenieria Electrica, Universidad Autonoma de Zacatecas, Zacatecas, 98160 ZAC, Mexico; ^4^Servicio de Endocrinologia Pediatrica, Hospital General “Gaudencio González Garza, ” Centro Medico Nacional La Raza, Instituto Mexicano del Seguro Social (IMSS), 02990 Ciudad de Mexico, Mexico; ^5^Unidad Academica de Enfermeria, Universidad Autonoma de Zacatecas, Zacatecas, 98160 ZAC, Mexico

## Abstract

Obesity is considered the main risk factor associated with the development of insulin resistance (IR). The aim of this study was to evaluate the influence of obesity on puberty onset and IR in Mexican children. A total of 378 children (189 boys and 189 girls) aged 8–14 years participated in the study. IR was estimated using the homeostasis model assessment for IR (HOMA-IR). The mean fasting glucose (FG) and basal insulin levels were 82 mg/dl and 11.0 *μ*IU/ml in boys and 77.3 mg/dl and 12.3 *μ*IU/ml in girls (*P* < 0.05). Subjects with obesity at Tanner stages II–V showed increased FG levels (*P* < 0.05). In boys with obesity, there was a decrease in HOMA-IR in Tanner stage IV and differences in age between boys with normal weight and those with obesity in Tanner V, being older the boys with obesity. Obesity in children and adolescents was associated with higher HOMA-IR values. In boys with obesity, IR increased at the end of pubertal maturation, with a delay in puberty. These findings should be considered on the establishment of IR cutoff values for pubertal population in Mexico and in the establishment of strategies to prevent the health problems related to obesity.

## 1. Introduction

Worldwide, obesity is one of the most important public health problems and is considered to be the greatest epidemic of the 21st century [1, 2]. In the juvenile population, a serious increase in the prevalence of obesity has also been documented [1, 2]. Mexico has the highest prevalence of childhood obesity in the world, according to statistics from the Organization for Economic Co-operation and Development (OECD) [1]. In 2016, the combined prevalence of overweight and obesity in Mexican children from 5 to 11 years old and in adolescents from 12 to 19 years old was calculated to be 33.2% and 36.3%, respectively [3].

Insulin resistance (IR) is a condition characterized by an attenuated response to the action of insulin, which results in decreased glucose uptake by muscle and adipose tissue cells, decreased hepatic glycogen production, and an increase in the production of hepatic glucose [4]. In the pediatric population, obesity is considered the main risk factor associated with the development of IR and, in turn, the IR is the most common metabolic disorder associated with obesity [4]. IR is observed in 50% of the children and adolescents with obesity [5] and is considered to be a common link and factor promoting the cascade of metabolic alterations observed in patients with obesity [6].

IR, with a compensatory high level of insulin in the blood and a family history of diabetes mellitus type 2 (DM2), plays important roles in the development of DM2 [7]. Accordingly, because DM2 is preceded by a period of IR, the measurement of both fasting insulin and glucose in children and adolescents with obesity is recommended, particularly in subjects who had the presence of acanthosis nigricans and a family history of DM2 [1, 2, 8–10]. There are several diagnostic methods for the determination of insulin sensitivity in the pediatric population; one of them is the HOMA (homeostasis model assessment) index [2], which is most commonly used in clinical practice because it is simple and has a high correlation with the hyperinsulinemic-euglycemic clamp, the gold standard technique for IR assessment [2, 9]. The existence of different HOMA cutoff values across populations is the main disadvantage of the technique, as this has complicated the selection, comparison, and therefore the establishment of a general consensus of suitable values for IR assessment. Several studies performed in adults have suggested a HOMA-IR cutoff value of 2.5 [11, 12]. In the pediatric population, especially during puberty, the HOMA-IR value is in general higher than that in adults. A cutoff value of 3.16 has been proposed by several population-based studies and is widely accepted by most authors [2, 8, 9, 13].

The HOMA index reflects insulin secretion and its hepatic and peripheral sensitivity [14]. There are several physiological conditions that increase IR, including puberty, pregnancy, and old age [15–17]. Puberty, defined as the period of human development during which physical growth and sexual maturity occurs [18], is characterized by a pronounced decrease in insulin sensitivity and a compensatory elevation in insulin secretion [16, 19]. Other changes associated with puberty include high levels of insulin growth factor 1 (IGF-1), growth hormone (GH), and sexual hormones [19, 20]. Some stages of pubertal maturation are associated with an increase in insulin secretion and the HOMA index, and it is generally expected that these alterations will return to normal values at the end of puberty [21]. In children with obesity the IR increases considerably, especially in girls [22]. The presence of variations in IR throughout life, with periods of physiological IR during puberty, complicates the interpretation and the assessment of the clinical significance of IR in children. Considering the high rate of child obesity in Mexico and the absence of association data related to obesity and IR in puberty and knowing that the early identification of IR in children could be useful to delay or prevent the onset of irreversible IR-associated pathological entities, the objective of this study was to evaluate the influence of obesity on puberty and IR in Mexican children.

## 2. Materials and Methods

### 2.1. Study Population

This prospective case-control study was performed in Zacatecas, Mexico. It was conducted according to the guidelines laid down in the Declaration of Helsinki and the protocol was approved by the Institutional Review Board of Universidad Autonoma de Zacatecas (Approval ID: BIOÉTICA-ACS/UAZ Proy. number 002/2014). The participants were drawn from 719 children aged 8–14 from eight selected public schools including elementary (4th–6th degree) and middle schools (7th–9th degree), from May 2014 to March 2015 (Figure S1). The conformation of the study groups was divided into two stages. First, detailed information related to the protocol was provided to the participants and his/her parent or guardian and written informed consent was obtained. All the participants who provided signed informed consent underwent a physical examination and a completed questionnaire on the risk factors for DM2 development. In the second stage, of all the evaluated participants, children with obesity according to Mexican guidelines (NOM-008-SSA3–2010) [23] were identified and included in the case group. The control group was formed of normal-weight children paired with case children according to gender and pubertal stage. The exclusion criteria of the study were the presence of comorbidities such as diabetes mellitus or hypertension. Accordingly, a total of 378 children were selected for the study. The case group was constituted of 185 children with obesity (boys: 97; girls: 88) and the controls included 193 children with normal weight (boys: 92; girls 101).

### 2.2. Biological Samples and Measurements

The participants included in the study groups (cases and controls) donated 2 ml of a fasting blood sample. The blood sample was obtained using tubes without an anticoagulant and processed at the Central Laboratory at Universidad Autonoma de Zacatecas. Serum glucose was measured by the glucose oxidase method using a Vitros™ DT II (Ortho Clinical Diagnostics, USA). Serum insulin levels were measured on an IMMULITE™ 1000 (Siemens Healthcare Diagnostics, Tarrytown, NY) using a solid-phase, two-site chemiluminescent immunometric assay (Siemens Healthcare Diagnostics, Tarrytown, NY). BMI was calculated as weight (kg) divided by height squared (m^2^). General obesity was defined as BMI ≥ 95th percentile of the sex-specific age-standardized BMI of the study population using Centers for Disease Control and Prevention (CDC) growth charts [23, 24]. Pubertal Tanner staging was performed by a specialist in pediatric endocrinology according to criteria published by Marshall and Tanner in 1969 [13, 25].

HOMA-IR was derived from fasting glucose and fasting insulin using an established formula: HOMA-IR = fasting glucose (mmol/lt) × fasting insulin (*μ*IU/ml)/22.5 [1]. Hyperglycemia was defined as fasting glucose ≥ 100 mg/dl [26]. Before the clinic visit, parents were instructed to have their children fast for at least 10 h.

### 2.3. Statistical Analysis

Comparisons of the risk factors and the clinical and personal characteristics among the groups were performed using the chi-squared or Fisher's exact test for categorical variables. Evaluations of continuous variables including simple comparisons between obese and control groups were performed using Student's *t*-test or the Mann–Whitney rank sum test. Differences in the clinical measurements among the different Tanner stages were evaluated using Kruskal-Wallis one-way analysis of variance (ANOVA) on ranks coupled with Dunn's method as a multiple comparison procedure. Univariate analysis was performed with Sigma Plot v.11 software (SYSTAT Software Inc., San Jose, CA). To evaluate the ability of all clinical and personal characteristics to predict HOMA-IR status, a multivariate analysis was carried out. Two feature selection approaches were performed; first, a bidirectional feature selection using Akaike information criterion (AIC) [27], followed by a fast backward variable selection using a method based on the study of Lawless [28]. These analyses were performed in R project, R Statistical Software [29]. For AIC selection, MASS package version 7.3-45 [30] was used and RMS package version 5.1-0 was used for fast backward variable selection [31]. Differences were considered of statistical significance at *P* < 0.05.

## 3. Results

A total of 378 children (189 boys and 189 girls) were selected for the study. The general characteristics of the study population are shown in Table 1 and supplementary Table S1.

The median age was 12.2 years for boys and 10.8 years for girls. Overall, 52.4% of boys and 47.6% girls had obesity. The mean of the fasting glucose level was higher (82.0 versus 77.3 mg/dl) and the mean of the fasting insulin level was lower in boys than in girls (11.0 versus 12.3*μ*IU/ml). Family history of DM2 was present in 72.4% and 76.4% of the children with obesity and with normal weight, respectively. Acanthosis nigricans was detected in an 85.9% of the children with obesity and in 36% of the children with normal weight (*P* < 0.001). The mean of HOMA-IR was 2.3 in the cohort whereas that for children with obesity was 3.23 and 1.5 for children with normal weight. 40% of the obese children had a HOMA-IR above 3.16. As expected, the proportions of Tanner staging were not different between groups of children with obesity and normal weight (*P* = 0.662) or between groups of girls and boys (*P* = 0.668).

**Figure 1:**
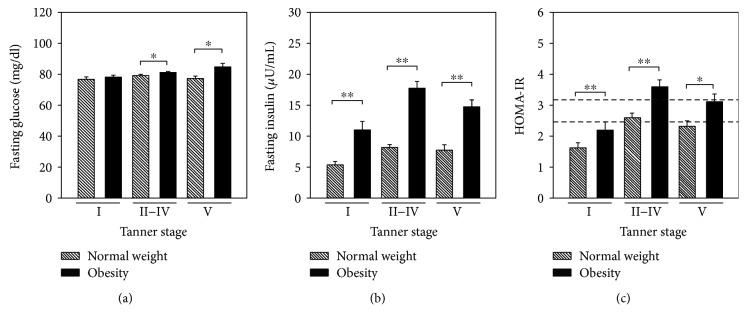
General comparisons of the clinical findings between study groups classified by Tanner stage. The study population was divided according Tanner stage (*y*-axis)—obesity (black bars) and normal-weight (diagonally striped bars) groups. Considering the normal-weight children as reference, fasting glucose (a), fasting insulin (b), and HOMA-IR (c) values were compared between groups at each Tanner stage using Student's *t*-test. Significant differences are shown at the top of the bar with an asterisk. ^∗^ *P* < 0.05 and ^∗∗^ *P* < 0.001. Dashed lines in the HOMA-IR plot indicate the cutoff values of 3.16 (dashed top line) and 2.15 (dashed bottom line).

Figure 1 shows the distribution of fasting glucose, fasting insulin, and HOMA-IR according with the Tanner stage in the study population. Considering the normal-weight children as reference, children with obesity in the Tanner stages II–IV and Tanner stage V showed higher fasting glucose levels (*P* < 0.05). There were differences in fasting insulin concentrations and HOMA-IR values between children with obesity and children with normal weight in each Tanner stage evaluated (Figures 1(b) and 1(c)); these were higher in the group of children with obesity (*P* < 0.001).

**Figure 2:**
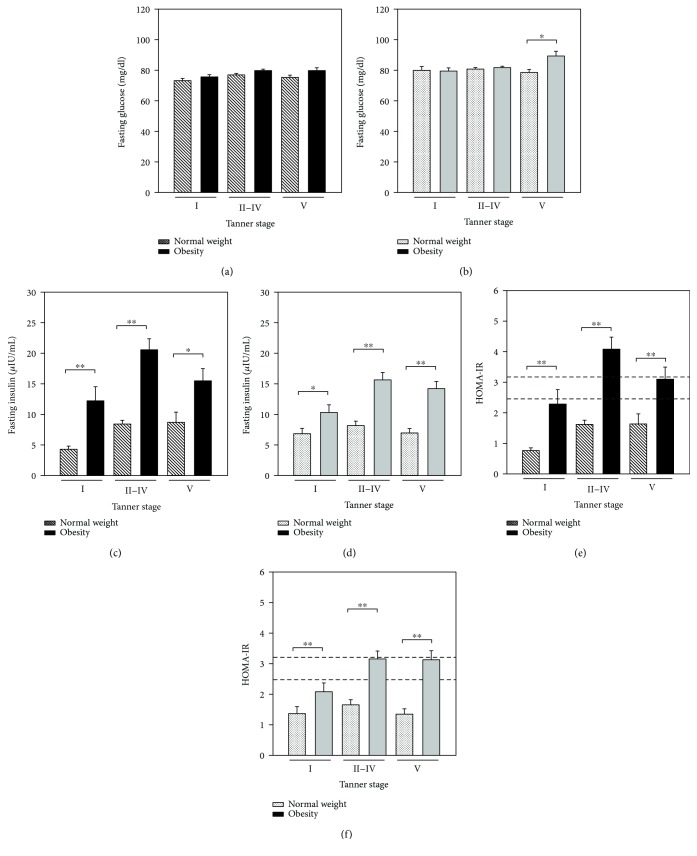
Comparison of clinical findings between study groups classified by sex and Tanner stage. Boys and girls were separated, and each group was subdivided according their Tanner stage (abscise axis)—obesity and normal-weight groups. Considering the normal-weight group as reference, fasting glucose, fasting insulin, and HOMA-IR values were compared between groups of girls (a, c, e) and boys (b, d, f) at each Tanner stage. Significant differences are shown at the top of the bar with an asterisk. ^∗^ *P* < 0.05 and ^∗∗^ *P* < 0.001. Dashed lines in HOMA-IR plot indicates the cutoff values of 3.16 (dashed top line) and 2.15 (dashed bottom line).

The insulin levels and HOMA-IR values were higher in the groups of girls and boys in Tanner stages II–IV (Figure 2). Classified by sex, both in boys and girls, there were differences in fasting insulin and HOMA-IR between normal weight and obesity in all the Tanner stages (*P* < 0.05). In boys in Tanner stage V, the mean fasting glucose concentration was different between the obese (89.8 mg/dl) and normal weight (79.1 mg/dl) groups (*P* < 0.05).

**Figure 3:**
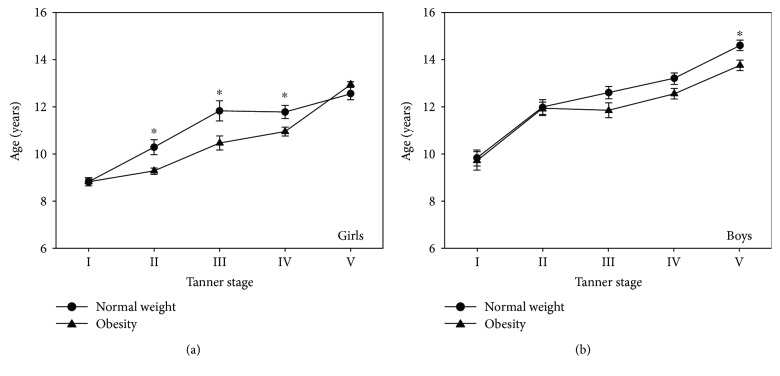
Comparison of the ages of the study groups classified by sex and Tanner stage. Boys and girls were separated, and each group was subdivided according their Tanner stage (*x*-axis)—obesity and normal-weight groups. Considering the normal-weight group as a reference, the ages in years (*y*-axis) were compared between groups of girls (a) and boys (b) at each Tanner stage. Significant differences are shown at the top of the bar with an asterisk.

To evaluate the effect of obesity on the sexual maturation of the study population, the girls and boys were grouped according to the different Tanner stages and their obesity or normal-weight status and their ages by Tanner stage were compared.  Figure 3 shows the results obtained from those comparisons. In the group of girls in Tanner stages II–IV, a difference in the mean age was found between girls with obesity and girls with normal weight (11.3 versus 10.4 years; *P* = 0.001). In the group of boys in Tanner stage V, a difference in the mean age between groups of children with obesity (14.6 years) and normal weight (13.7 years) was also observed (*P* = 0.007).

**Figure 4:**
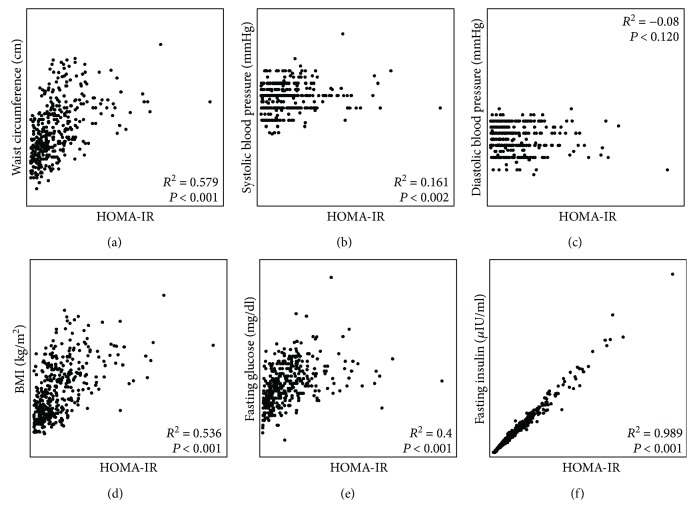
Correlation analysis between clinical features and HOMA-IR values. To evaluate the relationship between HOMA-IR (*x*-axis) and clinical findings (*y*-axis), a Pearson correlation analysis was performed. The figure shows the results obtained for the correlation studies between HOMA-IR and waist circumference (a), systolic blood pressure (b), diastolic blood pressure (c), BMI (d), fasting glucose (e), and fasting insulin (f). Correlation coefficients (*R* ^2^) and *P* values are shown in the plots.

To evaluate the relationship between pairs of clinical features of the study population, a correlation analysis was performed. The results are shown in Figure 4. There was a positive correlation between HOMA-IR and waist circumference, BMI, systolic blood pressure (SBP), fasting glucose, and fasting insulin (*P* < 0.05). As expected, a correlation coefficient close to 1 was observed for the relationship between HOMA-IR and fasting insulin (*R* ^2^ = 0.989; *P* < 0.001).

Once univariate models were compared, a multivariate analysis was carried out to evaluate the behavior of all clinical and personal characteristics to predict the status of HOMA-IR. A total of 317 children (168 boys and 149 girls) were selected for the multivariate study. These children had nonmissing values for the general characteristics included in this study. Fasting glucose and fasting insulin were removed to promote an unbiased multivariate search, given that HOMA-IR is calculated using both characteristics and thus is highly correlated. First, a bidirectional feature selection using AIC, which selects features that comprise an efficient multivariate model founded in an information theory, was used. AIC applied in both directions (i.e., forward and backward selection), provided a final model comprised of eight characteristics: gender, age, family history of DM2, DBP, SBP, acanthosis nigricans, BMI, and height, with a final AIC of 298.25 (Table 2).

**Figure 5:**
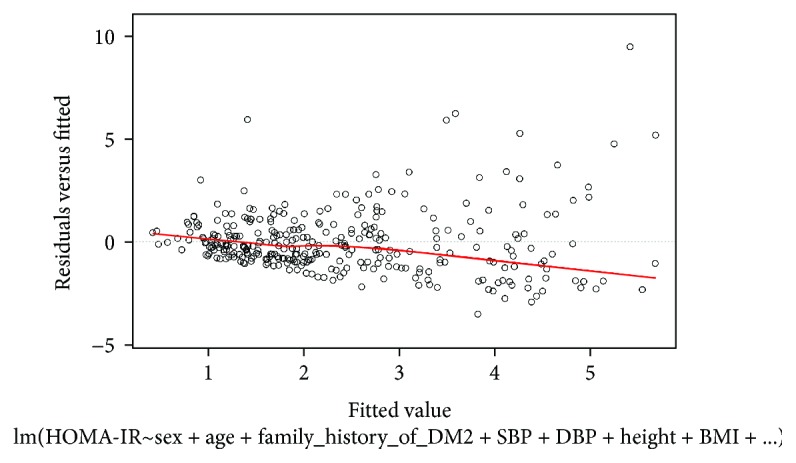
Residuals of the eight characteristic models.

Figure 5 shows the behavior of the model in terms of residuals, with an average of 1.085 and a median of 0.826 (*P* < 2.2 × 10^−16^).

Finally, a fast backward variable selection method using the *P* value with a significance level of 0.05 as an evaluation criterion for staying in the model was carried out. This method uses a conditional maximum likelihood to estimate the importance of each feature. Five characteristics survived the selection process: family history of DM2, DBP, height, acanthosis nigricans, and BMI.  Table 3 shows the *P* values associated with the eliminated characteristics. With this five-feature model, an absolute residual average of 1.102 and a median of 0.79 (*P*<2.2 × 10^−16^) were acquired.

## 4. Discussion

Obesity is considered to be a complex multifactorial disease that involves genetic, metabolic, biochemical, environmental, cultural, psychosocial, and lifestyle factors in its etiology [32]. It has been reported that children with obesity with more abdominal fat are more likely to become adults with obesity and that overweight and obesity increase the risk for pathological conditions such as prediabetes, DM2, high blood pressure, hypercholesterolemia, asthma, arthritis, cardiovascular, and poor health status [33, 34]. Accordingly, and considering the high rates of obesity in Mexican children as well as the absence of association data related to obesity and IR in puberty, in this study, the influence of obesity on puberty and IR was evaluated. Our results identified in the group with obesity SBP values significantly higher to those observed in the normal-weight group. In the same sense, the proportion of pubertal children with acanthosis nigricans, the most common dermatologic manifestation of obesity and hyperinsulinism, was higher in the obese participants; these findings are in agreement with previous reports [35].

One of the most concerning complications of childhood obesity is IR. In clinical practice, HOMA-IR is used to diagnose IR and is an independent predictor of cardiac pathology in adulthood [11]. A degree of IR is influenced by age, gender, race/ethnicity, the stage of sexual development, total adiposity, and fat distribution [36]. At present, there is no universally accepted pediatric definition for IR. The identification of IR children and adolescent is highly important as the occurrence of DM2 coincides with the peak of pubertal IR [37]. The interpretation of the HOMA-IR value is particularly challenging during adolescence. Several HOMA-IR cutoff values to define IR in adolescence have been suggested [12]. This study assessed IR by HOMA-IR in urban Mexican children and adolescents establishing a cutoff value of 3.16 for HOMA-IR, as suggested by Kurtoğlu et al. [11]. However, it has been reported that the HOMA-IR value increases with the age and pubertal stage of children and adolescents; for that reason, some authors prefer to use higher values [9]. Factors such as the development of puberty and ethnic differences are associated with variations in HOMA-IR from 1.8, 2.5, 3.16, and 3.2 to greater than 4 according to the population [2, 38]. In our study, the group with obesity showed higher values with statistical significance in fasting glucose, fasting insulin, HOMA-IR, and HOMA-IR > 3.16, with respect to their controls at any stage of pubertal development. In our study, 40% of the group with obesity had a HOMA-IR above 3.16, although this proportion is lower than the 60.4% reported by Ortega-Cortes et al. [1]; the differences between studies may be explained because, in the Ortega-Cortes study, the cutoff value used to determine IR was fixed as HOMA-IR ≥ 3.0 [1], reflecting the need to establish a consensus regarding HOMA-IR values for the Mexican prepubertal and pubertal populations.

Puberty is a complex process that consists of a series of predictable events, and the sequence of changes in secondary sexual characteristics has been categorized by staging systems. The most frequently utilized is the Tanner scale, which categorizes puberty into five stages [13, 25]. In our study, classifying the groups by Tanner stage, there were significant differences in the distribution of fasting glucose between Tanner stages II–V, fasting insulin, and HOMA-IR, both at each Tanner stage evaluated between children with obesity and normal weight and between groups of girls and boys (see Figures 1 and 2). According to previous reports, in healthy children, the IR is basal at Tanner stage I (prepubertal), increases at Tanner stage II (onset of puberty), peaks at Tanner stage III, and returns to near prepubertal levels at Tanner stage V (end of puberty) [34]. In our study population, the normal-weight group showed that the IR peaks at Tanner stage IV in both female and male subjects; this different pattern for HOMA-IR by Tanner stage could be explained in part to race and ethnicity [36]. At this point, it is important to note that, in the group of boys with obesity, a nonexpected HOMA-IR pattern was observed throughout the Tanner stages, presenting the IR peak at Tanner stage II with a decrease to near prepubertal levels at Tanner stage IV and then an increase at Tanner stage V (see Supplementary Figure S2); this is an interesting finding in our study. Moreover, in the last Tanner stage, boys with obesity showed a significant (close to one year) delay in puberty onset. In this respect, recent data suggest that excess adiposity during childhood may influence pubertal development as well. Specifically, excess adiposity during childhood may advance puberty in girls and delay puberty in boys [19]. Obesity in peripubertal boys may also be associated with reduced reproductive function, probably due to hypothalamic disturbances. Mechanisms explaining the effect of obesity on male fertility include abnormal reproductive hormone levels, increased release of adipose-derived hormones and adipokines associated with obesity, and other physical problems including sleep apnea and increased scrotal temperatures [39]. In our study, the correlation between pairs of clinical features of the study population was positive between HOMA-IR and waist circumference, BMI, SBP, fasting glucose, and fasting insulin, in accordance with previous reports [40].

Finally, some study limitations must be highlighted. In this study, the inclusion of a group of children and adolescents with overweight was not considered, and therefore, the behavior of IR in children with obesity, who are overweight, and with normal weight was not possible. In the same sense, although one of the study exclusion criteria included children/adolescents with prediabetes, DM, metabolic syndrome, and/or other concomitant diseases, future studies should be conducted to establish HOMA-IR comparisons in a broader range of pathologies.

## 5. Conclusions

Children and adolescents with obesity have higher HOMA-IR values than their counterparts with normal weight. In boys with obesity, IR increased at the end of pubertal maturation (Tanner V), with a delay in puberty. These findings should be considered in future studies focused on the establishment of IR cutoff values for children and adolescents in Mexico and/or in the establishment of strategies to prevent the health problems related to obesity.

## Figures and Tables

**Table 1:** Characteristics of study participants.

Characteristic	Group			*P* value
	General (*n* = 378)	Children with obesity (*n* = 185)	Normal weight (*n* = 193)	
Gender				
Female, *n* (%)	189 (50)	88 (47.6)	101 (52.3)	0.41
Male, *n* (%)	189 (50)	97 (52.4)	92 (47.7)	
Birth weight (g)	3099.6 ± 623.7	3131.4 ± 6	3069.9 ± 633.1	0.827
Weight (kg)	52.6 ± 17.7	64.2 ± 16.0	41.4 ± 10.6	<0.001
Height (m)	1.5 ± 0.1	1.56 ± 0.13	1.5 ± 0.14	<0.001
Waist circumference (cm)	79.0 ± 14.4	89.8 ± 11.4	68.6 ± 7.8	<0.001
Body mass index (kg/m^2^)	22.1 ± 4.9	26.2 ± 3.6	18.2 ± 2.0	<0.001
Diastolic blood pressure (mmHg)	76.3 ± 11.6	76.4 ± 11.7	76.2 ± 11.5	0.966
Systolic blood pressure (mmHg)	108.5 ± 11.4	110.9 ± 11.0	106.1 ± 11.3	<0.001
Family history of DM2, *n* (%)	282 (74.6)	134 (72.4)	148 (76.4)	0.406
Acanthosis nigricans, *n* (%)	195 (51.6)	159 (85.9)	36 (18.7)	<0.001
Tanner stage				
1	72 (19.0)	35 (18.9)	37 (19.2)	0.662
2	73 (19.3)	41 (22.2)	32 (16.6)	
3	81 (21.4)	37 (20.0)	44 (22.8)	
4	80 (21.2)	36 (19.5)	44 (22.8)	
5	72 (19.0)	36 (19.5)	36 (18.7)	
Fasting glucose (mg/dl)	79.6 ± 8.4	81.1 ± 8.6	78.2 ± 7.9	0.001
Fasting insulin (*μ*IU/mL)	11.7 ± 9.2	15.9 ± 10.6	7.6 ± 4.9	<0.001
HOMA-IR	2.3 ± 1.9	3.23 ± 2.19	1.5 ± 1.1	<0.001
^∗^HOMA-IR > 3.16, *n* (%)	87 (23.0)	74 (40.0)	13 (6.7)	<0.001

^∗^HOMA-IR cutoff value for pediatric population.

**Table 2:** Final feature selection with AIC.

Feature	Degrees of freedom	Sum of squares	RSS	AIC
None			745.89	289.25
−sex	1	5.695	751.59	289.66
−family history of DM2	1	6.161	752.05	289.86
+waist circumference	1	2.165	743.73	290.33
+birth weight	1	1.300	744.59	290.70
+weight	1	1.200	744.69	290.74
+obesity (presence/absence)	1	0.747	745.15	290.93
+Tanner stage	1	0.651	745.24	290.97
−age	1	8.944	754.84	291.03
−height	1	11.641	757.53	292.16
−systolic blood pressure	1	13.621	759.51	292.99
−diastolic blood pressure	1	24.440	770.33	297.47
−body mass index	1	26.122	772.01	298.16
−acanthosis nigricans	1	61.472	807.36	312.36

HOMA-IR~sex + age + family history of DM2 + SBP + DBP + height + BMI + acanthosis nigricans. AIC = 289.25.

**Table 3:** Eliminated features using the fast-backward approach.

Deleted	Chi-square	Degrees of freedom	*P* value	Residual	Degrees of freedom	*P* value	AIC
Tanner stage	0.00	1	0.979	0.00	1	0.979	−2.00
Waist circumference	1.07	1	0.300	1.07	2	0.585	−2.93
Sex	0.89	1	0.346	1.96	3	0.581	−4.04
Obesity (presence/absence)	1.83	1	0.176	3.80	4	0.434	−4.20
Systolic blood pressure	3.1	1	0.078	6.89	5	0.229	−3.11
Birth weight	3.05	1	0.081	9.94	6	0.127	−2.06
Age	2.99	1	0.084	12.93	7	0.074	−1.07
Weight	3.43	1	0.064	16.36	8	0.038	0.36

## Data Availability

The data used to support the findings of this study are available from the corresponding author upon request.

## Supplementary Materials

Supplementary Materials Figure S1: location of the schools participating in the study. The study population (*n* = 378) was recruited from three elementary (*n* = 171) and five middle (*n* = 207) schools from the cities of Zacatecas and Guadalupe, Mexico; 189 girls (♀) and 189 boys (♂). Scale 1 : 2000 ft. Table S1: characteristics of study participants classified by sex. Figure S2: comparison between clinical findings between study groups classified by gender and specific Tanner stages.
