# Supplementary material for: The Influence of Obesity on Puberty and Insulin Resistance in Mexican Children
Source: Int J Endocrinol. 2018 Sep 3;2018:7067292. doi: 10.1155/2018/7067292 (PMC6140127; doi:10.1155/2018/7067292)
Supplement: Supplementary Materials — Figure S1: location of the schools participating in the study. The study population (n = 378) was recruited from three elementary (n = 171) and five middle (n = 207) schools from the cities of Zacatecas and Guadalupe, Mexico; 189 girls (♀) and 189 boys (♂). Scale 1 : 2000 ft. Table S1: characteristics of study participants classified by sex. Figure S2: comparison between clinical findings between study groups classified by gender and specific Tanner stages. [file 7067292.f1.docx]

**- Supplementary Information -**

**The influence of obesity on puberty and insulin resistance in Mexican children**

Edith Cardenas-Vargas,^1,2^ Jairo A. Nava,^2^ Idalia Garza-Veloz,^2,3^ Mayra C. Torres-Castañeda,^4^ Carlos E. Galván-Tejada,^3^ Miguel A. Cid-Baez,^2^ Rosa E. Castañeda-Arteaga,^1,2^ Yolanda Ortiz-castro,^2^ Perla M. Trejo-Ortiz,^5^ Roxana Araujo-Espino,^5^ Fabiana E. Mollinedo-Montaño,^5^ Jose Ramon Muñoz-Torres,^2^ and Margarita L. Martinez-Fierro^2,3^

^1^Hospital General Zacatecas "Luz González Cosío", Servicios de Salud de Zacatecas, Zacatecas 98160, Mexico.

^2^Molecular Medicine Laboratory, Unidad Academica de Medicina Humana y C.S, Universidad Autonoma de Zacatecas, Zacatecas 98160, Mexico.

^3^Unidad Academica de Ingenieria Electrica, Universidad Autonoma de Zacatecas, Zacatecas 98160, Mexico.

^4^Servicio de Endocrinologia Pedriatrica. Hospital General “Gaudencio González Garza”. Centro Medico Nacional La Raza. Instituto Mexicano del Seguro Social (IMSS). Ciudad de Mexico 02990, Mexico.

^5^Unidad Academica de Enfermeria, Universidad Autonoma de Zacatecas, Zacatecas 98160, Mexico.

To whom the correspondence should be addressed:

Margarita L Martinez-Fierro, D.Sc.

Unidad Academica de Medicina Humana y Ciencias de la Salud.

Universidad Autonoma de Zacatecas.

Carretera Zacatecas-Guadalajara Km.6. Ejido la Escondida, Zacatecas, Mexico.

e-mail: [margaritamf@uaz.edu.mx](mailto:margaritamf@uaz.edu.mx).

*And*

Edith Cardenas-Vargas. MD.

Departamento de Enseñanza e Investigacion. Hospital General Zacatecas "Luz González Cosío", Servicios de Salud de Zacatecas, Zacatecas, Mexico. Circuito Ciudad Gobierno No. 410. Col. Ciudad Gobierno. C.P. 98160. Tel.: +52 (492) 4914130

E-mail address: [edithcardenasv@gmail.com](mailto:edithcardenasv@gmail.com)

**
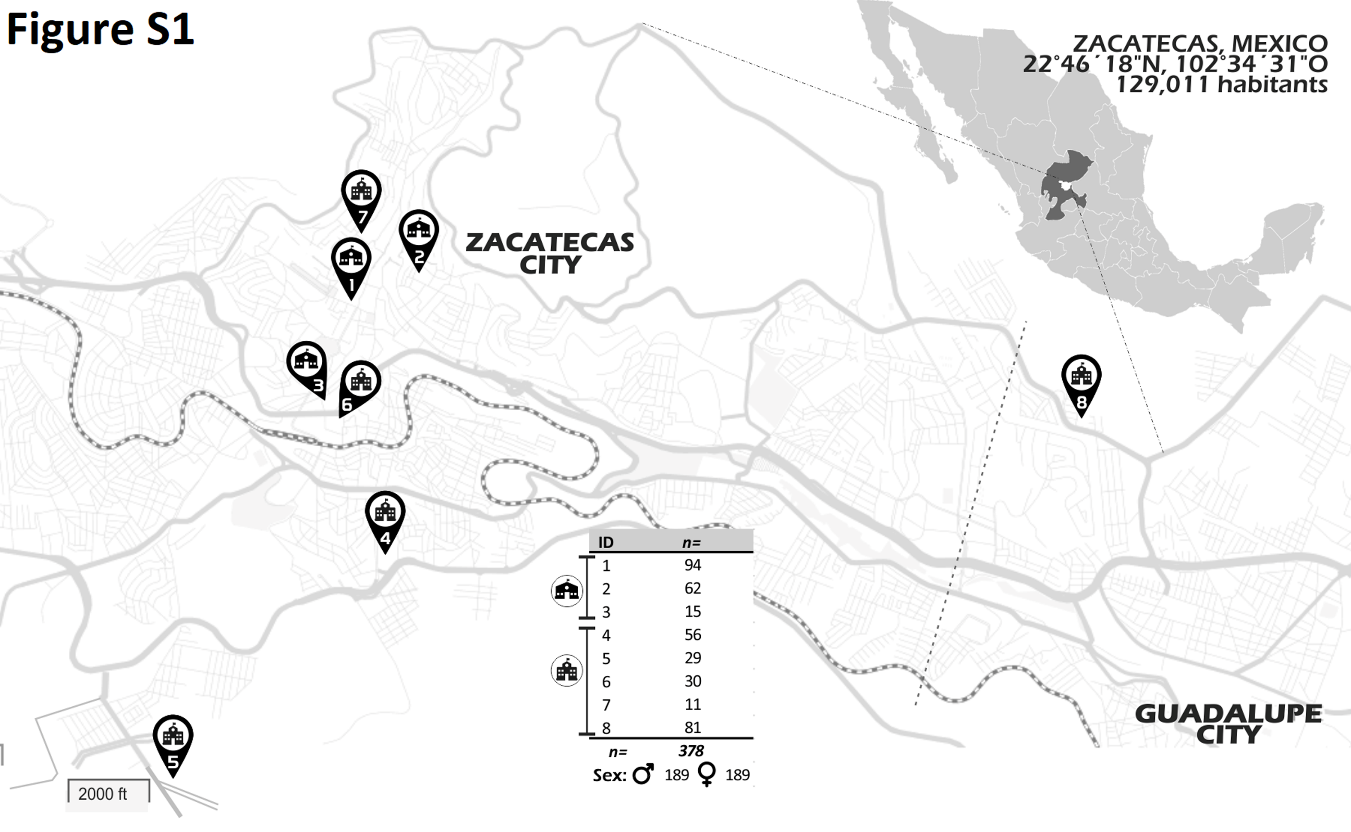
**

Figure S1: Location of the schools participating in the study. The study population (n = 378) was recruited from three elementary (n = 171) and five middle schools (n = 207) from the cities of Zacatecas and Guadalupe, Mexico; 189 girls (♀) and 189 boys (♂). Scale 1:2000 ft.

| **Table S1.** Characteristics of study participants classified by sex | | | |  |
| --- | --- | --- | --- | --- |
| **Characteristic** | **General**  **(n= 378)** | **Girls**  **(n=189)** | **Boys**  **(n=189)** | ***P-*value^†^** |
| Age (years) | 11.5 ± 1.9 | 10.8 ± 1.8 | 12.2 ± 1.9 | **< 0.001** |
| Birthweight (g) | 3099.6 ± 623.7 | 3015.8 ± 616.9 | 3194.1 ± 619.9 | 0.009 |
| Weight (kg) | 52.6 ± 17.7 | 48.5 ± 16.0 | 56.7 ± 18.4 | **< 0.001** |
| Height (m) | 1.5 ± 0.1 | 1.5 ± 0.1 | 1.6 ± 0.1 | **< 0.001** |
| Waist circumference (cm) | 79.0 ± 14.4 | 75.4 ± 12.9 | 82.6 ± 14.9 | **< 0.001** |
| Body mass index (kg/m^2^) | 22.1 ± 4.9 | 21.6 ± 4.8 | 22.5 ± 5.0 | 0.081 |
| Diastolic blood pressure (mmHg) | 76.3 ± 11.6 | 73.8 ± 11.7 | 78.8 ± 10.9 | **< 0.001** |
| Systolic blood pressure (mmHg) | 108.5 ± 11.4 | 105.6 ± 10.8 | 111.3 ± 11.2 | **< 0.001** |
| Family history of DM2, n (%) | 282 (74.6) | 145 (76.7) | 137 (72.5) | 0.408 |
| Acanthosis nigricans, n (%) | 195 (51.6) | 81 (42.9) | 114 (60.3) | **< 0.001** |
| Tanner stage |  |  |  |  |
| 1 | 72 (19.0) | 37 (51.0) | 35 (49.0) | 0.668 |
| 2 | 73 (19.3) | 31 (42.0) | 42 (58.0) |  |
| 3 | 81 (21.4) | 44 (54.0) | 37 (46.0) |  |
| 4 | 80 (21.2) | 41 (51.0) | 39 (49.0) |  |
| 5 | 72 (19.0) | 36 (50.0) | 36 (50.0) |  |
| Fasting glucose (mg/dl) | 79.6 ± 8.4 | 77.3 ± 7.3 | 82.0 ± 8.8 | **< 0.001** |
| Fasting insulin (μIU/mL) | 11.7 ± 9.2 | 12.3 ± 10.5 | 11.0 ± 7.7 | 0.59 |
| HOMA-IR | 2.3 ± 1.9 | 2.4 ± 2.1 | 2.3 ± 1.7 | 0.82 |
| *HOMA-IR > 3.16, n (%) | 87 (23.0) | 45 (23.8) | 42 (22.2) | 0.807 |
| ^†^*P* value from the comparison between girls and boys. Data are expressed as mean ± SD or frequency (%). *HOMA-IR cutoff value for the pediatric population. | | | | |


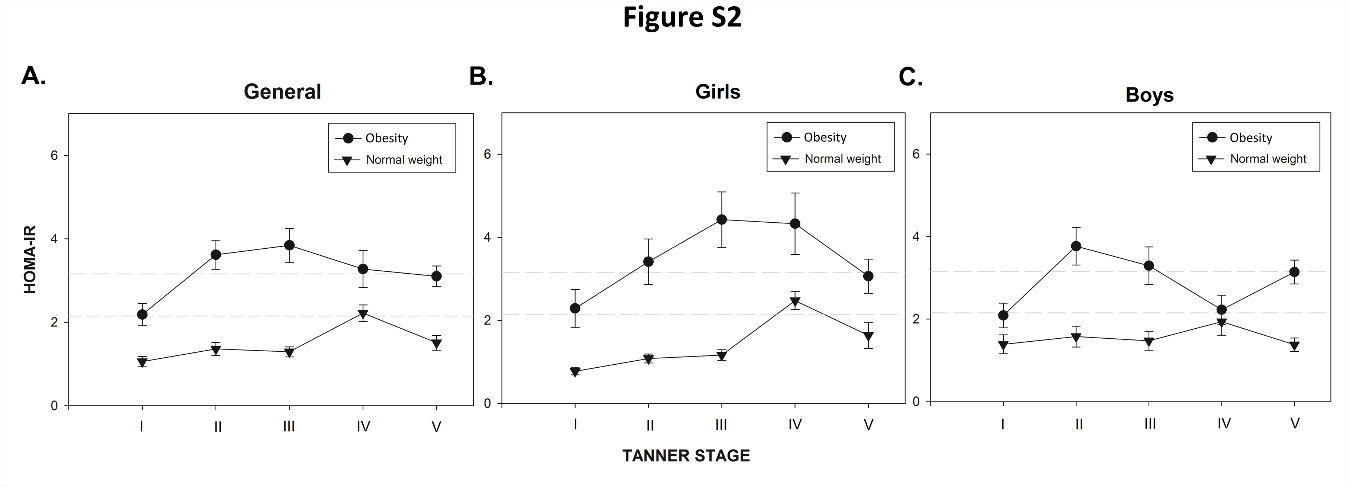


**Figure S2:** Comparison between clinical findings between study groups classified by gender and specific Tanner stages.
